# The Use of Two-Dimensional Strain Measured by Speckle Tracking in the Identification of Incipient Ventricular Dysfunction in HIV-Infected Patients on Antiretroviral Therapy, Untreated HIV Patients and Healthy Controls

**DOI:** 10.5935/abc.20190169

**Published:** 2019-10

**Authors:** Ronaldo Campos Rodrigues, Katia Martins Lopes de Azevedo, Samuel Datum Moscavitch, Sergio Setubal, Claudio Tinoco Mesquita

**Affiliations:** Universidade Federal Fluminense (UFF), Niterói, RJ - Brazil

**Keywords:** Acquired Immunodeficiency Syndrome, HIV, Ventricular Disfunction,Left, Echocardiography, Doppler, Antiretroviral Therapy, Highly Active, Strain, Speckle Tracking

## Abstract

**Background:**

Most cardiovascular abnormalities in patients infected with the human immunodeficiency virus (HIV) have been associated with myocardial damage directly caused by the virus. Some cases, however, may be associated with adverse effects from antiretroviral therapy (ART). New ventricular function assessment techniques are capable of detecting early changes in the cardiac function of HIV-infected patients using or not using ART. The usefulness of these techniques has been little employed in these patients.

**Objectives:**

To investigate the potential influence of antiretroviral therapy (ART) on the occurrence of subclinical left ventricular systolic dysfunction evaluated by myocardial strain rate analysis using two-dimensional speckle tracking echocardiography (2-D Echo) in treated HIV patients compared to untreated patients and healthy individuals.

**Methods:**

Sixty-eight HIV-infected patients with no cardiovascular symptoms, normal left ventricular (LV) ejection fraction (> 0.55 on 2-D Echo) were divided into three groups: 11 patients not using antiretroviral therapy (NT), 24 using protease inhibitor (PI) and 33 using non-nucleoside reverse transcriptase inhibitor (NNRTI). We also studied 30 normal non-HIV infected individuals (Ctrl). Demographic, clinical, biochemical and anthropometric data were collected. Preliminary transthoracic echocardiography included study of myocardial strain using two-dimensional speckle tracking. We studied strain and strain rate in the seventeen left ventricular (LV) myocardial segments in the longitudinal, circumferential and radial axes. Statistical analysis of the data was done with IBM SPSS - version 20 for Windows. Upon analysis of the data, namely the normality of independent variables in the different groups and the homogeneity of the variances between the groups, Kruskal-Wallis’ non-parametric test was done, followed by Dunn’s multiple comparison tests to test the significance of the differences between the values measured in the study groups. A significance level of 5% was adopted for decision-making on statistical tests.

**Results:**

The mean age of HIV patients was 40 ± 8.65 years and the mean age of controls was 50 ± 11.6 years (p < 0.001). Median LV global longitudinal strain (GLS) of NT patients (-17.70%), PI patients (-18.27%) and NNRTIs (-18.47%) were significantly lower than that of the Ctrl group (-20.77%; p = 0.001). There was no significant difference in mean SLG between treated patients (PI, NNRTI) and untreated (NT) patients. No significant differences were observed in mean circumferential and radial strain, nor on circumferential and radial strain rates between the NT, PI, NNRTI and Ctrl groups.

**Conclusion:**

The data suggest that HIV patients present, on myocardial strain measured by speckle tracking, signs of early LV systolic dysfunction that seem to be unrelated to the presence of ART. The prognostic significance of this condition in these patients deserves further studies.

## Introduction

Around the world, deaths related to the acquired immunodeficiency syndrome (AIDS) declined from about 1.9 million in 2005 to about 1 million in 2016, largely due to expansion of treatment - for the first time, more than half of people infected with the human immunodeficiency virus (HIV) were under treatment for the disease. Since 2010, the annual number of new infections in all age groups decreased by 16%. However, progress is variable and, despite a global downward trend in this epidemic disease, several regions have been experiencing a sharp increase in the number of new infections and difficulties in expanding treatment.^1^

The antiretroviral therapy (ART) was an important development for HIV-infected patients, contributing to prolonged survival and improved quality of life.^2^ Cardiovascular diseases have become a common finding because of the longer survival of these patients. Another important aspect of cardiovascular complications is that they appear to be associated with the effects of ART.^3,4^ Although a decline in the incidence of severe heart conditions due to opportunistic agents, malnutrition or prolonged immunosuppression has been observed,^5^ the incidence of coronary artery disease and peripheral vascular events has increased in HIV-infected patients.^6,7^

HIV-infected patients may have specific myocardial abnormalities and conventional two-dimensional tests may fail to detect subtle abnormalities in regional myocardial function. Speckle tracking is an innovative echocardiographic technique that has the capacity to evaluate myocardial strain in order to identify subtle abnormalities in ventricular function. Myocardial strain is a very important mechanical variable in HIV-infected patients, as it shows subclinical left ventricular dysfunction. Unfortunately, the technique of studying cardiac strain is still underused. Global longitudinal strain (GLS) is well correlated with left ventricular ejection fraction (LVEF). Reduced SLG can be found in patients with heart failure with preserved ejection fraction,^8^ stable angina,^9^ three-vessel coronary artery disease and patients using chemotherapy agents with cardiotoxicity.^10,11^ The purpose of this study was to evaluate the presence of subclinical ventricular function abnormalities in HIV-infected patients using or not using ART.

## Methods

Observational cross-sectional study involving 68 HIV-infected patients recruited from the Infectiology Service of Hospital Universitário Antônio Pedro (HUAP), Universidade Federal Fluminense (UFF). Inclusion criteria were: age ≥18 years, HIV infection confirmed by serological tests, no cardiovascular symptoms. Patients were excluded if they were under any therapy with cardiac or neurological medications, if they had any cardiac symptom or history of hypertension, LV ejection fraction <0.55 and pulmonary artery systolic pressure >36 mmHg, stable angina, atrial fibrillation or moderate to severe valvular heart disease. Echocardiography was performed as part of an established research protocol rather than for symptoms or comorbidities. Patients were divided into four groups: 1) HIV-positive patients not using ART (NT); 2) HIV-positive patients on protease inhibitor therapy for at least 12 months (PI); 3) HIV-positive patients on therapy with non-nucleoside reverse transcriptase inhibitors (NNRTI) for at least 12 months and 4) healthy controls. Samples from the NT (n = 11), PI (n = 24) and NNRTI (n = 33) groups were defined by convenience, considering the patients at the time of data collection. For the control group, a sample of size similar to the largest of the study groups (n = 30) was defined.

**Figure 1:**
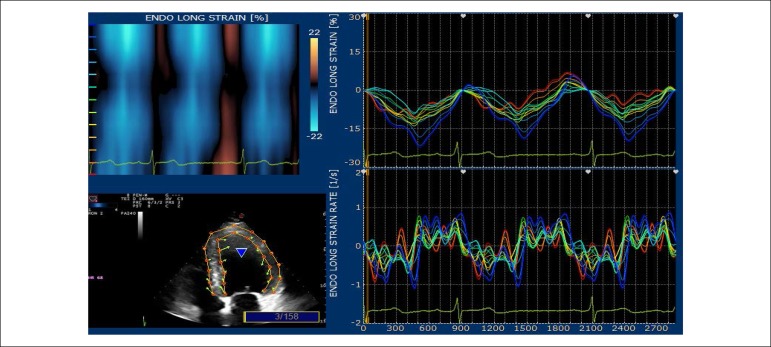
Apical four-chamber – percentage of longitudinal strain in the basal, middle and apical segments of the inferior and anterolateral septal walls.

**Figure 2:**
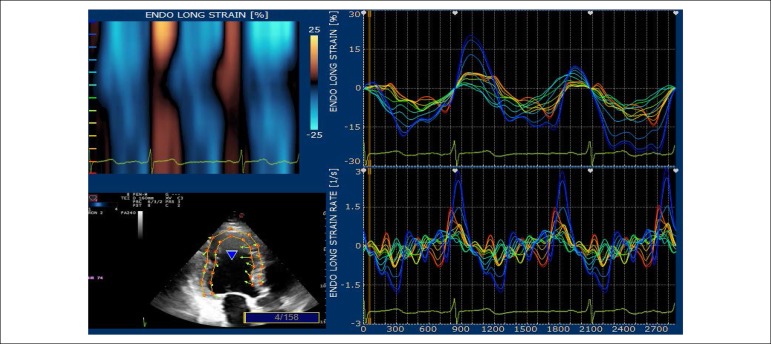
Apical three-chamber – percentage of longitudinal strain in the basal, middle and apical segments of the inferolateral and anterior septal walls.

**Figure 3:**
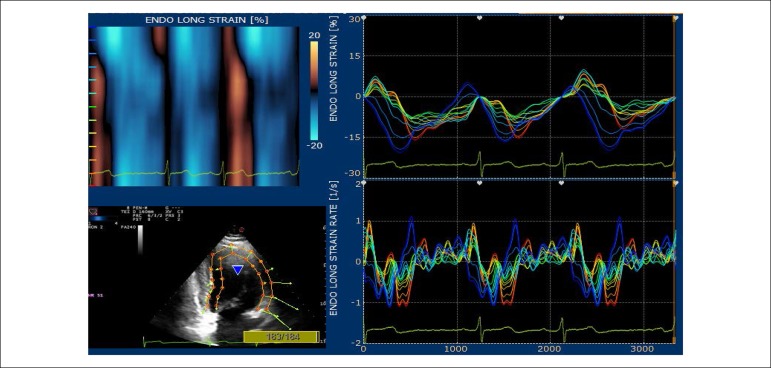
Apical two-chamber – percentage of longitudinal strain in the basal, middle and apical segments of the inferior and anterior walls.

The echocardiographic tests were conducted on an Echo Color Doppler device of the Italian company Esaote Biomédica, model Mylab 30 Gold, with a multi-frequency electronic sectoral transducer (2 to 4 MHz) with continuous electrocardiographic scanning. Traditional measures of left ventricular (LV) systolic function, ejection fraction and systolic shortening, diastolic function indicators, such as mitral flow E/A ratio, myocardial E wave velocity in the septal mitral annulus (septal E’), E/E’ ratio and estimated left atrial pressure were taken. Right ventricular diastolic diameter and two echocardiographic variables that evaluate right ventricular systolic function were determined: tissue Doppler of lateral tricuspid annulus and longitudinal tricuspid annular motion (LTAM). LV ejection fraction was determined by using the Simpson’s technique, on apical four-chamber and two-chamber views, on diastole and systole, thus obtaining end diastolic and end systolic volumes. Left atrial volume was obtained from end-systolic four-chamber and two-chamber views, and the arithmetic mean was then indexed by the body surface area to obtain left atrial volume index. LV mass was obtained from diastolic and systolic LV diameters, as well as from the interventricular septal and inferolateral wall diastolic thickness, following the technical guidelines of the American Society of Echocardiography.^12^ Maximum tricuspid regurgitation (TR) rate, an indicator of pulmonary artery pressure, was obtained from apical four-chamber view. LV diastolic and systolic myocardial velocities were obtained by placing the tissue Doppler sample volume in the septal mitral annulus. Digital myocardial strain curves were taken by using the Xstrain software package from scanned cross-sectional and apical view images. Myocardial strain rate was also evaluated. GLS was obtained by the arithmetic mean of the longitudinal strain values in the seventeen segments, from the four-chamber apical view (Figure 1), three-chamber apical view (Figure 2) and two-chamber apical view (Figure 3). Global circumferential strain (GCS) was obtained by the arithmetic mean of the circumferential strain values in the seventeen segments, from the cross-sectional views at the level of the mitral valve, papillary muscles and tip. Radial global strain (SRG) was obtained from the arithmetic mean of the radial strain values in the seventeen segments, from cross-sectional views of the mitral valve, papillary muscles and tip. Strain percentage analysis was repeated twice, using the best echocardiographic images. The same echocardiographer conducted transthoracic evaluation, then took the scanned images to calculate the percentages of longitudinal, radial and circumferential strain on an offline workstation. The strain rate in the longitudinal, circumferential and radial planes was also obtained. (Figures 1 and 3).

### Statistical analysis

Statistical analysis of the data was done with IBM SPSS - version 20 for Windows. After analysis of normality of independent variables in the different groups (using the Shapiro-Wilk test) and homogeneity of the variances between the groups (using Levene’s test), it was decided to use Kruskal-Wallis’ non-parametric test followed by Dunn’s multiple comparison tests to test the significance of the differences between the values measured in the study groups. A significance level of 5% was adopted for decision-making on statistical tests. Continuous variables with normal distribution were described as mean and standard deviation and continuous variables with non-normal distribution were described as median and interquartile range.

This study was approved by the Research Ethics Committee from Hospital Universitário Antônio Pedro (#HUAP 159/11) and all patients signed an Informed Consent Form.

## Results

The study included 98 individuals: 68 (69.4%) HIV-infected and 30 (30.6%) healthy controls with negative serology, of which 60 (61.2%) were males and 38 (38.8%) were females. Separately analyzing the groups of HIV-infected patients, 55.8% were males and 44,2% were females. The demographic, laboratory and clinical characteristics of the study population are found in table 1.

**Table 1:** Demographic, clinical and laboratory variables according to the group

Variable		NT (n = 11)	PI (n = 24)	NNRTI (n = 33)	Control (n = 30)	Kruskal-Wallis test (p)
Age (years)	M ± DP	33.3 ± 4.1	44.8 ± 8.7	39.4 ± 8.0	49.9 ± 11.6	< 0.001
	Md ± IQR	33.0 ± 3.0	43.0 ± 10.5	39.0 ± 8.0	48.5 ± 15.0	
Gender	Male	9 (81.8%)	15 (62.5%)	14 (42.4%)	22 (73.3%)	0.033 (chi-square)
	Female	2 (18.2%)	9 (37.5%)	19 (57.6%)	8 (26.7%)	
	Md ± IQR					
Heart rate (bpm)	M ± SD	75.6 ± 7.3	72.0 ± 8.9	78.1 ± 6.2	73.2 ± 6.1	0.029
	Md ± IQR	78.0 ± 14.0	73.5 ± 16.0	77.0 ± 9.5	75.0 ± 8.8	
SBP (mmHg)	M ± SD	121.4 ± 6.0	129.3 ± 6.8	129.1 ± 6.8	123.8 ± 5.5	0.001
	Md ± IQR	120.0 ± 5.0	130.0 ± 10.0	130.0 ± 5.0	125.0 ± 10.0	
DBP (mmHg)	M ± SD	71.4 ± 4.5	70.0 ± 7.7	70.3 ± 7.5	67.8 ± 5.7	0.338
	Md ± IQR	70.0 ± 5.0	70.0 ± 20.0	70.0 ± 15.0	70.0 ± 5.0	
Blood glucose (mg/dl)	M ± SD	84.8 ± 14.2	79.7 ± 11.2	83.0 ± 9.5	82.4 ± 5.7	0.455
	Md ± IQR	82.0 ± 23.0	79.0 ± 9.0	81.0 ± 13.5	81.0 ± 7.0	
Total cholesterol (mg/dL)	M ± SD	164.6 ± 26.8	189.0 ± 56.3	183.9 ± 30.0	196.3 ± 17.4	0.021
	Md ± IQR	163.0 ± 40.0	198.0 ± 54.0	181.0 ± 42.0	199.0 ± 26.0	
LDL-c (mg/dL)	M ± SD	102.6 ± 27.6	108.7 ± 48.5	110.0 ± 27.9	118.9 ± 18.5	0.229
	Md ± IQR	104.0 ± 49.0	109.0 ± 66.0	102.0 ± 36.0	122.0 ± 27.0	
HDL-c (mg/dL)	M ± SD	47.5 ± 16.7	42.6 ± 17.5	55.0 ± 16.0	53.4 ± 3.5	0.007
	Md ± IQR	43.0 ± 30.0	41.0 ± 22.0	52.0 ± 18.0	54.0 ± 4.0	
Triglycerides (mg/dL)	M ± SD	101.1 ± 39.1	174.8 ± 78.2	119.7 ± 115.2	127.0 ± 15.5	< 0.001
	Md ± IQR	86.0 ± 79.0	165.0 ± 106.0	87.0 ± 71.0	123.5 ± 27.0	
CD4+ lymphocytes/mm^3^	M ± SD	502.5 ± 206.3	534.4 ± 323.1	693.2 ± 317.7	-	0.044
	Md ± IQR	426.0 ± 310.0	404.0 ± 436.0	644.0 ± 297.0	-	

M: mean; SD: standard deviation; Md: median; IQR: interquartile range; BMI: body mass index; SBP: systolic blood pressure; DBP: diastolic blood pressure; NT: HIV-positive patients not using antiretroviral therapy. PI: HIV-positive patients on protease inhibitor therapy. NNRTI: HIV-positive patients on non-nucleoside reverse transcriptase inhibitor therapy. Control: healthy HIV-negative individuals.

The age range was 27 to 81 years (43.26 ± 10.58 years). There were 34 individuals in the age group of 27 to 37 years, 37 individuals in the age group of 38 to 48 years, 19 individuals in the age group of 49 to 59 years, 6 individuals in the age group of 60 to 70 years and 2 individuals in the age group of 71 to 81 years.

Table 1 shows the demographic, clinical and laboratory variables of the different groups. Table 2 shows the echocardiographic variables of the different groups.

**Table 2:** Echocardiographic variables

Variable		NT (n = 11)	PI (n = 24)	NNRTI (n = 33)	Control (n = 30)	Kruskal-Wallis test (p)
Aorta (mm)	M ± SD	28.27 ± 1.85	30.58 ± 3.02	28.73 ± 2.97	29.53 ± 2.08	0.026
	Md ± IQR	29.00 ± 3.00	30.00 ± 3.50	29.00 ± 4.00	30.00 ± 2.00	
LA diameter (mm)	M ± SD	31.18 ± 3.82	33.21 ± 3.22	31.64 ± 4.59	34.33 ± 2.55	0.004
	Md ± IQR	30.00 ± 6.00	32.00 ± 4.50	31.00 ± 3.00	34.00 ± 4.00	
LVDd-i (mm/m^2^)	M ± SD	29.75 ± 0.79	29.75 ± 1.81	28.67 ± 2.36	28.20 ± 1.73	0.020
	Md ± IQR	29.70 ± 1.13	29.87 ± 2.75	29.73 ± 4.22	28.65 ± 2.84	
LVSD (mm)	M ± SD	30.36 ± 2.38	31.17 ± 4.04	32.18 ± 3.26	32.17 ± 2.78	0.248
	Md ± IQR	30.00 ± 5.00	31.00 ± 7.00	32.00 ± 4.00	31.50 ± 4.00	
IVS (mm)	M ± SD	7.18 ± 0.98	7.88 ± 1.08	7.91 ± 0.95	9.03 ± 0.76	< 0.001
	Md ± IQR	7.00 ± 2.00	8.00 ± 2.00	8.00 ± 2.00	9.00 ± 2.00	
PP (mm)	M ± SD	7.00 ± 1.00	7.42 ± 1.18	7.67 ± 0.92	8.33 ± 0.80	< 0.001
	Md ± IQR	7.00 ± 0.00	8.00 ± 1.00	8.00 ± 1.00	9.00 ± 1.00	
LVEF – Simpson (%)	M ± SD	66.64 ± 3.83	62.46 ± 3.60	63.55 ± 4.10	64.17 ± 3.50	0.030
	Md ± IQR	67.00 ± 5.00	62.00 ± 5.00	63.00 ± 6.00	64.00 ± 6.00	
LV mass index (g/m^2^)	M ± SD	82.23 ± 16.76	104.49 ± 24.01	90.01 ± 19.54	108.12 ± 14.25	< 0.001
	Md ± IQR	82.28 ± 13.59	106.35 ± 37.69	89.34 ± 26.89	110.61 ± 18.10	
E/A ratio	M ± SD	1.46 ± 0.40	1.33 ± 0.34	1.52 ± 0.41	1.18 ± 0.07	< 0.001
	Md ± IQR	1.34 ± 0.38	1.30 ± 0.22	1.50 ± 0.44	1.18 ± 0.09	
E’ septal annulus (cm/s)	M ± SD	9.55 ± 1.87	9.03 ± 1.91	10.54 ± 2.21	8.38 ± 0.41	< 0.001
	Md ± IQR	9.00 ± 1.90	8.35 ± 2.00	10.00 ± 3.00	8.15 ± 0.60	
S’ septal annulus (cm/s)	M ± SD	8.25 ± 1.09	8.10 ± 0.68	8.49 ± 1.41	9.03 ± 0.95	0.001
	Md ± IQR	8.00 ± 2.00	8.00 ± 0.40	8.10 ± 1.00	8.80 ± 0.60	
E/E’ ratio	M ± SD	8.41 ± 1.33	8.72 ± 2.03	7.08 ± 1.65	9.29 ± 0.62	< 0.001
	Md ± IQR	8.40 ± 2.59	9.08 ± 3.04	7.27 ± 2.45	9.38 ± 0.88	
LA volume index (ml/m²)	M ± SD	30.38 ± 6.16	29.93 ± 4.76	29.48 ± 5.60	29.56 ± 1.81	0.839
	Md ± IQR	29.11 ± 1.09	30.40 ± 3.48	29.17 ± 7.32	29.88 ± 2.76	
S lateral tricuspid annulus (cm/s)	M ± SD	11.29 ± 1.54	10.87 ± 1.42	12.23 ± 1.90	11.49 ± 0.90	0.014
	Md ± IQR	11.00 ± 2.00	10.80 ± 2.00	12.00 ± 2.00	11.70 ± 0.50	

M: mean; SD: standard deviation; Md: median; IQR: interquartile range; ST: HIV+ patient not using antiretroviral therapy. PI: HIV-positive patients on protease inhibitor therapy; NNRTI: HIV-positive patients on non-nucleoside reverse transcriptase inhibitor therapy. LVDD: left ventricular diastolic diameter; LVSD: left ventricular systolic diameter; ΔD%: left ventricle; LA: left atrium

Regarding the echocardiographic variable “LV mass indexed by BSA,” we identified higher values in groups HIV+ PI and HIV- CONTROL compared to groups HIV+ NO MEDICATION and HIV+ NNRTI. There were no differences between the groups HIV+ PI and HIV- CONTROL, nor among the groups HIV+ NO MEDICATION and HIV+ NNRTI (Table 2).

Regarding the variable “PP” (septal diastolic thickness), we identified higher values in the HIV-CONTROL GROUP. There were no differences between the groups HIV+ PI, HIV+ NO MEDICATION and HIV+ NNRTI (Table 2).

Regarding the variable “SIV” (posterior wall diastolic thickness), we identified higher values in the HIV-CONTROL GROUP. There were no differences between the groups HIV+ PI, HIV+ NO MEDICATION and HIV+ NNRTI, although the group HIV+ NO MEDICATION presented PP values lower than the others (Table 2).

### Global longitudinal strain

Table 3 shows the GLS in the different groups.

**Table 3:** Behavior of global longitudinal strain according to the group

Variable		NT (n = 11)	PI (n = 24)	NNRTI (n = 33)	Control (n = 30)	Kruskal-Wallis test (p)
Global longitudinal strain	M ± SD	-18.11 ± 1.28	-17.96 ± 4.89	-18.15 ± 3.07	-20.66 ± 0.79	0.001
	Md ± IQR	-17.70 ± 2.07	-18.27 ± 6.14	-18.47 ± 4.27	-20.77 ± 1.00	

M: mean; SD: standard deviation; Md: median; IQR: interquartile range.

Mean SLG was lower in the HIV groups compared to controls (p < 0.05). There were no differences between groups of HIV-infected patients.

No statistically significant differences were identified between longitudinal, circumferential and radial strain rates between the groups of HIV-infected patients and controls.

## Discussion

The purpose of our study was to identify subclinical left ventricular dysfunction using speckle tracking. HIV+ patients were asymptomatic from the cardiovascular point of view and had normal LV systolic function by conventional echocardiographic analysis based on LV ejection fraction.

This study demonstrated that patients with HIV infection, even those not on ART, present longitudinal myocardial strain abnormalities assessed by speckle tracking. These findings confirm previous observations^13^ and extend them by assessing the impact of new therapeutic protocols.

Cardiovascular manifestations of HIV infection were altered by the introduction of ART, which significantly modified the course of HIV infection, decreasing mortality and improving the quality of life of infected patients. On the other hand, data from multiple studies raised the concern that ART would be associated with an increase in peripheral and coronary artery disease. The clinical manifestations associated with ART are frequent and must be followed up by the multidisciplinary teams assisting these patients.^14^

This study suggests that subclinical left ventricular dysfunction should be investigated whenever possible. Speckle tracking is an advanced echocardiographic technique that has much greater sensitivity than transthoracic echocardiography to detect functional abnormalities, mainly cardiac strain variables that assess left ventricular mechanical efficiency, identifying abnormalities earlier than other imaging techniques.

Sims et al.,^15^ using transthoracic echocardiography, evaluated 28 HIV-infected young adults (aged seven to twenty-nine), compared to 28 controls, and no abnormalities of systolic and diastolic parameters were found. However, on the study of cardiac strain, a decrease in the percentage of longitudinal strain was observed in the patients in comparison with the control group. HIV-infected patients, regardless of ART, had a lower longitudinal strain rate than the control group.

Multiple studies have found high triglyceride levels in HIV-infected patients using protease inhibitors,^16-18^ as these drugs stimulate the synthesis of hepatic triglycerides.^19^ In our study, the group of patients using protease inhibitors presented the highest serum triglyceride levels. Studies in the literature show the importance of monitoring the lipid profile of HIV-infected patients using ART, especially when using protease inhibitors.^20^

In this study, it was observed that groups of HIV-infected patients, regardless of the type of ART, presented lower global longitudinal strain percentage than healthy controls. Barbaro et al.^3^ evidenced in their study the need to monitor this group of patients, seeking to identify individuals with higher cardiovascular risk.

Previous studies evaluated left ventricular systolic and diastolic function in the population of HIV-infected individuals using one-dimensional and two-dimensional echocardiography and spectral Doppler. Hsue et al.^21^ and Reinsch et al.^22^ studied left ventricular diastolic and systolic functions using tissue Doppler, which uses filters for high velocities (blood) obtaining systolic and diastolic myocardial velocities in the septal and lateral mitral annulus. Lang et al.^12^ focused their research on the complete study of LV diastolic function, following a scaled evaluation flowchart according to the guidelines of the American Society of Echocardiography.^12^ Others identified anatomical and functional abnormalities in infected patients on ART.^23-28^ The most recent studies use myocardial strain and myocardial strain rate percentage using speckle tracking to detect subclinical ventricular dysfunction in HIV-infected patients on ART.^12,29,30^

We know that the longitudinal cardiac fiber strain can be used to study the behavior of myocardial fibers arranged in the subendocardial area, as we know that 77% of these fibers are disposed longitudinally, and this makes speckle tracking play an important role in the study of ischemic disease, since ischemia begins in the subendocardial region.

This study revealed lower longitudinal strain percentages in HIV-infected individuals compared to healthy controls. There were no differences between the percentages of longitudinal strain in groups of HIV-infected patients using or not using ART.

Accurate and reproducible estimate of myocardial damage in patients with HIV infection and using ART has been considered to be increasingly important. The CHAART-2 study, which identified the long-term cardiovascular effects in HIV-infected children on ART, showed that cardiac structure and function were superior in HIV-infected children exposed to ART in the perinatal period compared with children in the pre-ART^31^ era, which demonstrates the importance of early treatment in preventing cardiac damage. Besides, it reinforces the need for monitoring cardiac function in HIV-infected patients using ART to identify early myocardial injury, thereby decreasing long-term cardiovascular complications.

Several published papers have demonstrated the relationship between AIDS and cardiovascular diseases, with pericardial effusion and pericarditis being the best known.^32-39^

Okoshi and Montenegro^40^ studied the incidence and etiology of heart lesions in patients with AIDS through a retrospective study of 72 necropsies. In none of the patients, death was considered a consequence of cardiac lesion, but macro and microscopic abnormalities were found in 90% of the cases.

Several studies report that the prevalence of cardiac abnormalities may be underestimated. Interstitial lymphocytic myocarditis^41,42^ is found in 50 to 70% of asymptomatic infected individuals.

Myocardial abnormalities appear to be associated with more severe cases of immunosuppression and low TCD4 counts.^43^

### Limitations

The limitations of the study are the limited sample size and the relatively broad age range of HIV-infected participants. Neither the effect of disease duration nor ART duration were analyzed. The absence of coronary artery disease documentation on computed tomography angiography did not allow to evaluate the influence of ART on the development of CAD. Currently, there are few patients not on antiretroviral therapy, therefore the group of HIV-infected patients not on ART was smaller than the other groups. We know that speckle tracking is a technique that depends on image quality and on the observer’s experience in evaluating the main curves of myocardial strain. We have observed a large number of studies using speckle tracking in an attempt to identify patients with subclinical left ventricular dysfunction, but we should increasingly stimulate further research with a greater number of investigated patients to better understand the significance of the findings in the prognosis of patients.

## Conclusion

The technique of studying myocardial strain by speckle tracking was able to detect early signs of deterioration of myocardial systolic function in HIV-infected patients, regardless of whether or not they were on antiretroviral drugs. Further studies are needed to evaluate HIV-infected patients and to assess the prognostic significance of these abnormalities in these patients.
